# Supplementary material for: A systems-level model reveals that 1,2-Propanediol utilization microcompartments enhance pathway flux through intermediate sequestration
Source: PLoS Comput Biol. 2017 May 5;13(5):e1005525. doi: 10.1371/journal.pcbi.1005525 (PMC5438192; doi:10.1371/journal.pcbi.1005525)
Supplement: S1 Text — This appendix elaborates on the model formulations in the Models section and on the equations used in the analytical and numerical code to generate the plots and other figures. (PDF) [file pcbi.1005525.s001.pdf]

Supplementary Information

To accompany Jakobson, Slininger, Tullman-Ercek, and Mangan; A Systems-Level Model Reveals That 1,2-Propanediol Utilization Microcompartments Enhance Pathway Flux Through Intermediate Sequestration.

The following describes the equations used in the numerical and analytical code used to generate the plots and other figures in the text. The codebase will be made available on GitHub following publication under a GNU General Public License.

Analytical solutions

We can find the following complete analytical solutions in the cytosol as a function of the concentrations in the MCP:

$$P(r) = \frac{k_m^P P_{MCP}(r = R_c) - P_{out}(j_c + k_m^P)}{\frac{D}{R_b^2} + k_m^p X} \left(\frac{1}{r} - \frac{D}{k_c^P R_c^2} - \frac{1}{R_c} \right) + P_{MCP}(r = R_c) \quad (1)$$

$$A(r) = \frac{A_{MCP}(r = R_c) - A_{out}}{\frac{D}{k_m^A R_b^2} + X} \left(\frac{1}{r} - \frac{D}{k_c^A R_c^2} - \frac{1}{R_c} \right) + A_{MCP}(r = R_c) \quad (2)$$

Where $X = \left(\frac{D}{R_c^2 k_c} + \frac{1}{R_c} - \frac{1}{R_b} \right)$

We can then use the solution in the cytosol to generate the following boundary condition at the MCP membrane:

$$\frac{\partial P}{\partial r}|_{r=R_c} = -\left(\frac{1}{R_c^2} \right) \frac{k_m^P P_{MCP}(r = R_c) - P_{out}(j_c + k_m^P)}{\frac{D}{R_b^2} + k_m^p X} \quad (3)$$

$$\frac{\partial A}{\partial r}|_{r=R_c} = -\left(\frac{1}{R_c^2} \right) \frac{A_{MCP}(r = R_c) - A_{out}}{\frac{D}{k_m^A R_b^2} + X} \quad (4)$$

First, consider the mass balance on A_{MCP} :

$$\int_0^{R_c} k_c^A (A_{cyt} - A_{MCP}) dA + \int_0^{R_c} R_{CDE} - R_{PQ} dV = 0 \quad (5)$$

$$4\pi R_c^2 k_c^A (A_{cyt} - A_{MCP}) + \frac{4}{3}\pi R_c^3 \left(\frac{V_{CDE} P_{MCP}}{K_{MCDE} + P_{MCP}} - \frac{2V_{PQ} A_{MCP}}{K_{MPQ} + A_{MCP}} \right) = 0 \quad (6)$$

And similarly for P_{MCP}:

$$\int_0^{R_c} k_c^P (P_{cyt} - P_{MCP}) dA - \int_0^{R_c} R_{CDE} dV = 0 \quad (7)$$

$$4\pi R_c^2 k_c^P (P_{cyt} - P_{MCP}) - \frac{4}{3}\pi R_c^3 \frac{V_{CDE} P_{MCP}}{K_{MCDE} + P_{MCP}} = 0 \quad (8)$$

We now assume that the concentrations in the MCP are constant since $\xi >> 1$. First we solve for P_{MCP}, as this does not depend on A_{MCP} due to the irreversibility of

PduCDE. We simplify the solution by defining the following important timescales, assuming that $k_c = k_c^a = k_c^p$ and $k_m = k_m^a = k_m^p$ (Table 2):

$$\tau_{cell}^{diff} = \frac{R_b^2}{D}; \tau_{MCP}^{diff} = \frac{R_c^2}{D}; \tau_{cell}^{trans} = \frac{R_b}{3k_m}; \tag{9}$$

$$\tau_{MCP}^{trans} = \frac{R_c}{3k_c}; \tau_{CDE} = \frac{K_{MCDE}}{V_{CDE}}; \tau_{PQ} = \frac{K_{MPQ}}{2V_{PQ}} \tag{10}$$

Letting $p = \frac{P_{MCP}}{K_{MCDE}}$, $\lambda = 1 + \frac{j_c}{k_m}$, $\rho = \frac{R_c}{R_b}$, and $p^* = \frac{P_{out}}{K_{MCDE}}$, the solution for p is therefore as follows:

$$\Gamma_{CDE} = \frac{\tau_{MCP}^{diff}}{\tau_{CDE}} \left(\frac{\tau_{cell}^{trans}}{\tau_{cell}^{diff}} + \frac{\tau_{MCP}^{trans}}{\tau_{MCP}^{diff}} + \frac{1}{3}\rho + \frac{1}{3} \right) \tag{11}$$

$$p = \frac{\lambda p^* - \Gamma_{CDE} - 1 \pm \sqrt{(1 - \lambda p^* + \Gamma_{CDE})^2 + 4\lambda p^*}}{2} \tag{12}$$

Furthermore, if PduCDE is saturated,

$$p = \lambda p^* - \Gamma_{CDE} \tag{13}$$

We can estimate the magnitudes of these various timescales based on the baseline model parameters (Table 2) and thence analyze the magnitude of the various terms in Γ_{CDE}.

$$\frac{\tau_{MCP}^{diff}}{\tau_{CDE}} \approx \frac{10^{-5}}{3x10^{-3}} = 3.33x10^{-3} \tag{14}$$

$$\frac{\tau_{cell}^{trans}}{\tau_{cell}^{diff}} \approx \frac{4x10^{-1}}{2.5x10^{-4}} = 1.6x10^{3} \tag{15}$$

$$\frac{\tau_{MCP}^{trans}}{\tau_{MCP}^{diff}} \approx \frac{3x10^{-1}}{10^{-5}} = 3x10^{4} \tag{16}$$

Therefore, for the baseline model parameter values in Table 1,

$$\Gamma_{CDE} \approx \frac{\tau_{MCP}^{diff}}{\tau_{CDE}} \left(\frac{\tau_{MCP}^{trans}}{\tau_{MCP}^{diff}} \right) = \frac{\tau_{MCP}^{trans}}{\tau_{CDE}} = O(10^2) \tag{17}$$

$$\lambda p^* \approx p^* = 10^2 \tag{18}$$

Suggesting that in the vicinity of the baseline parameter values, the solution for P in the MCP is governed by the relative timescales of the transport of 1,2-PD in and out of the MCP and the reaction of 1,2-PD to propionaldehyde by PduCDE, as well as by the external 1,2-PD concentration.

Now we can find A_{MCP} similarly, given the solution for P_{MCP}. Letting $a = \frac{A_{MCP}}{K_{MPQ}}$, $a^* = \frac{A_{out}}{K_{MPQ}}$ and $\Omega = \frac{V_{CDE}}{2V_{PQ}}$,

$$\Gamma_{PQ} = \frac{\tau_{MCP}^{diff}}{\tau_{PQ}} \left(\frac{\tau_{cell}^{trans}}{\tau_{cell}^{diff}} + \frac{\tau_{MCP}^{trans}}{\tau_{MCP}^{diff}} + \frac{1}{3}\rho + \frac{1}{3} \right) \tag{19}$$

$$a = \frac{a^* + \Gamma_{PQ}(\frac{p}{1+p}\Omega - 1) - 1 \pm \sqrt{(1 - a^* + \Gamma_{PQ}(1 - \Omega\frac{p}{1+p}))^2 + 4(\Omega\Gamma_{PQ}\frac{p}{1+p} + a^*)}}{2} \tag{20}$$

If PduCDE is saturated and A_{out} is negligible, then

$$a = \frac{\Gamma_{PQ}(\Omega - 1) - 1 \pm \sqrt{(1 + \Gamma_{PQ}(1-\Omega))^2 + 4\Omega\Gamma_{PQ}}}{2} \tag{21}$$

And if both PduCDE and PduPQ are saturated and A_{out} is negligible, then

$$a = \Gamma_{PQ}(\Omega - 1) \tag{22}$$

We can analyze the relative magnitudes of the timescales in Γ_{PQ} as above, assuming the baseline parameter values in Table 1, and we find that

$$\frac{\tau_{MCP}^{diff}}{\tau_{PQ}} \approx \frac{10^{-5}}{3x10^{-1}} = 3.33x10^{-5} \tag{23}$$

$$\Gamma_{PQ} \approx \frac{\tau_{MCP}^{diff}}{\tau_{PQ}} \left(\frac{\tau_{MCP}^{trans}}{\tau_{MCP}^{diff}} \right) = \frac{\tau_{MCP}^{trans}}{\tau_{PQ}} = O(1) \tag{24}$$

$$\Omega = O(1) \tag{25}$$

$$a^* = 0 \tag{26}$$

Suggesting that in the vicinity of the baseline parameter values, the solution for A in the MCP is governed by the relative timescales of the transport of propionaldehyde in and out of the MCP and the reaction of propionaldehyde by PduP/Q, as well as by the relative rates of PduCDE and PduP/Q.

Again, the solutions in the cytosol follow directly from these MCP solutions.

Governing equations for computation

As described in the Models section, the equations describing the concentrations of P and A in the MCP are as follows:

$$D\nabla^2 P(r) - R_{CDE} = 0 \tag{27}$$

$$D\nabla^2 A(r) + R_{CDE} - R_{PQ} = 0 \tag{28}$$

And the concentrations in the cytosol are described by the following:

$$P(r) = \frac{k_m^P P_{MCP}(r = R_c) - P_{out}(j_c + k_m^P)}{\frac{D}{R_b^2} + k_m^p X} \left(\frac{1}{r} - \frac{D}{k_c^P R_c^2} - \frac{1}{R_c} \right) + P_{MCP}(r = R_c) \tag{29}$$

$$A(r) = \frac{A_{MCP}(r = R_c) - A_{out}}{\frac{D}{k_m^A R_b^2} + X} \left(\frac{1}{r} - \frac{D}{k_c^A R_c^2} - \frac{1}{R_c} \right) + A_{MCP}(r = R_c) \tag{30}$$

Where $X = \left(\frac{D}{R_c^2 k_c} + \frac{1}{R_c} - \frac{1}{R_b} \right)$

The following boundary conditions hold at the cell and MCP membranes, respectively:

$$D\frac{\partial P}{\partial r}|_{r=R_b} = j_c P_{out} + k_m^P \left(P_{out} - P_{cytosol}(r = R_b) \right) \tag{31}$$

$$D\frac{\partial A}{\partial r}|_{r=R_b} = k_m^A \left(A_{out} - A_{cytosol}(r = R_b)\right) \tag{32}$$

$$\frac{\partial P}{\partial r}|_{r=R_c} = -\left(\frac{1}{R_c^2}\right)\frac{k_m^P P_{MCP}(r = R_c) - P_{out}(j_c + k_m^P)}{\frac{D}{R_b^2} + k_m^p X} \tag{33}$$

$$\frac{\partial A}{\partial r}|_{r=R_c} = -\left(\frac{1}{R_c^2}\right)\frac{A_{MCP}(r = R_c) - A_{out}}{\frac{D}{k_m^A R_b^2} + X} \tag{34}$$

Non-dimensional equations

We then recast the system in terms of the following non-dimensional variables:

$$\rho = \frac{r}{R_c} \tag{35}$$

$$a = \frac{A}{K_{PQ}} \tag{36}$$

$$p = \frac{P}{K_{CDE}} \tag{37}$$

Applying the non-dimensionalization and letting $\kappa = \frac{K_{CDE}}{K_{PQ}}$, we obtain the following governing equation for A in the MCP:

$$\frac{K_{PQ}D}{R_c^2}\frac{1}{\rho^2}\frac{\partial}{\partial \rho}\left(\rho^2\frac{\partial a}{\partial \rho}\right) = \frac{2V_{PQ}a}{1+a} - \frac{V_{CDE}p}{1+p} \tag{38}$$

Now let $\gamma = \frac{2V_{PQ}}{V_{CDE}}$ and $\xi = \frac{K_{PQ}D}{V_{CDE}R_c^2}$, yielding:

$$\xi\frac{1}{\rho^2}\frac{\partial}{\partial \rho}\left(\rho^2\frac{\partial a}{\partial \rho}\right) = \xi\nabla^2 a = \gamma\frac{a}{1+a} - \frac{p}{1+p} \tag{39}$$

Similarly for P in the MCP,

$$\kappa\xi\frac{1}{\rho^2}\frac{\partial}{\partial \rho}\left(\rho^2\frac{\partial p}{\partial \rho}\right) = \kappa\xi\nabla^2 p = \frac{p}{1+p} \tag{40}$$

We can then nondimensionalize the boundary conditions as follows:

$$\frac{\partial a}{\partial \rho}|_{\rho=1} = \epsilon_a + \beta_a a \tag{41}$$

$$\epsilon_a = \frac{A_{out}}{R_c K_{PQ}\left(\frac{D}{k_m^A R_b^2} + X\right)} \tag{42}$$

$$\beta_a = \frac{-1}{R_c\left(\frac{D}{k_m^A R_b^2} + X\right)} \tag{43}$$

Similarly for P,

$$\frac{\partial p}{\partial \rho}|_{\rho=1} = \epsilon_p + \beta_p p \tag{44}$$

$$\epsilon_p = \frac{P_{out}\left(j_c + k_m^P\right)}{K_{CDE}R_c\left(\frac{D}{R_b^2} + k_m^P X\right)} \tag{45}$$

$$\beta_p = \frac{-k_m^P}{R_c\left(\frac{D}{R_b^2} + k_m^P X\right)} \tag{46}$$

These nondimensional equations can then be solved numerically by a finite-difference approach to find the steady-state concentrations in the MCP, and the solutions in the cytosol follow directly. We solve the spherical finite-difference equations using the `ODE15s` solver in MATLAB.

Analytical solution

For ease of computation, we cast the analytical solution (assuming constant concentrations in the MCP) differently than in the Models section.

First, consider the mass balance on A_{MCP} :

$$0 = 4\pi R_c^2 k_c^A (A_{cyt} - A_{MCP}) + \frac{4}{3}\pi R_c^3 (\frac{V_{CDE} P_{MCP}}{K_{CDE} + P_{MCP}} - \frac{2V_{PQ} A_{MCP}}{K_{PQ} + A_{MCP}}) \tag{47}$$

And similarly for P_{MCP}:

$$0 = 4\pi R_c^2 k_c^P (P_{cyt} - P_{MCP}) - \frac{4}{3}\pi R_c^3 \frac{V_{CDE} P_{MCP}}{K_{CDE} + P_{MCP}} \tag{48}$$

First we solve for P_{MCP}, as this does not depend on A_{MCP} due to the irreversibility of PduCDE:

$$k_c^P (P_{cyt} - P_{MCP}) = \frac{1}{3} R_c \frac{V_{CDE} P_{MCP}}{K_{CDE} + P_{MCP}} \tag{49}$$

$$\frac{3DK_{CDE}}{V_{CDE} R_c^3 (\frac{D}{k_m^P R_b^2} + X)} \Big[\frac{P_{out}(1 + \frac{j_c}{k_m^P})}{K_{CDE}} - p \Big] = \frac{p}{1+p} \tag{50}$$

Let $Y = \frac{V_{CDE} R_c^3 (\frac{D}{k_m^P R_b^2} + X)}{3DK_{CDE}}$ and let $Z = \frac{P_{out}(1+\frac{j_c}{k_m^P})}{K_{CDE}}$.

$$\frac{1}{Y}(Z - p) = \frac{p}{1+p} \tag{51}$$

Let $E = Y - Z + 1$.

$$p = \frac{-E \pm \sqrt{E^2 + 4Z}}{2} \tag{52}$$

Now we can find A_{MCP} similarly, given the solution for P_{MCP}.

$$\frac{3D}{R_c^3 (\frac{D}{k_m^A R_b^2} + X)} (A_{out} - A_{MCP}) = \frac{2V_{PQ} A_{MCP}}{K_{PQ} + A_{MCP}} - \frac{V_{CDE} P_{MCP}}{K_{CDE} + P_{MCP}} \tag{53}$$

$$\frac{3DK_{PQ}}{2V_{PQ} R_c^3 (\frac{D}{k_m^A R_b^2} + X)} (\frac{A_{out}}{K_P Q} - a) + \frac{1}{2} \frac{V_{CDE}}{V_{PQ}} \frac{p}{1+p} = \frac{a}{1+a} \tag{54}$$

Let $U = \frac{2V_{PQ} R_c^3 (\frac{D}{k_m^P R_b^2} + X)}{3DK_{PQ}}$, let $V = \frac{A_{out}}{K_{PQ}}$, and let $W = \frac{1}{2}\frac{V_{CDE}}{V_{PQ}}$.

$$\frac{1}{U}(V - a) + W \frac{p}{1+p} = \frac{a}{1+a} \tag{55}$$

Let $F = 1 + U - V$.

$$a = \frac{-(F - UW\frac{p}{1+p}) \pm \sqrt{(F - UW\frac{p}{1+p})^2 + 4(UW\frac{p}{1+p} + V)}}{2} \tag{56}$$

Again, the solutions in the cytosol follow directly from these MCP solutions.

Equations for no MCP case

In the case when there is no Pdu MCP, we assume that the same number of enzymes are now distributed throughout the cell. The equations in the cell are therefore now as follows:

$$0 = D\nabla^2 A + R_{CDE} - R_{PQ} \tag{57}$$

$$0 = D\nabla^2 P - R_{CDE} \tag{58}$$

These can be non-dimensionalized as follows (*c.f.* with above for MCP case):

$$\xi \frac{1}{\rho^2}\frac{\partial}{\partial \rho}\left(\rho^2 \frac{\partial a}{\partial \rho}\right) = \xi \nabla^2 a = \gamma \frac{a}{1+a} - \frac{p}{1+p} \tag{59}$$

Similarly for P,

$$\kappa\xi \frac{1}{\rho^2}\frac{\partial}{\partial \rho}\left(\rho^2 \frac{\partial p}{\partial \rho}\right) = \kappa\xi \nabla^2 p = \frac{p}{1+p} \tag{60}$$

Now considering the boundary conditions,

$$\frac{\partial A}{\partial r}|_{r=R_b} = k_m^A (A_{out} - A) \tag{61}$$

$$\frac{\partial a}{\partial \rho}|_{\rho=1} = \epsilon_a + \beta_a a \tag{62}$$

$$\epsilon_a = \frac{R_b A_{out} k_m^A}{DK_{PQ}} \tag{63}$$

$$\beta_a = \frac{-R_b k_m^A}{D} \tag{64}$$

Similarly for P,

$$\frac{\partial p}{\partial \rho}|_{\rho=1} = \epsilon_p + \beta_p p \tag{65}$$

$$\epsilon_p = \frac{R_b P_{out}}{DK_{CDE}}(j_c + k_m^P) \tag{66}$$

$$\beta_p = \frac{-R_b k_m^P}{D} \tag{67}$$

These equations can once again be solved numerically by the same finite difference approach described above.
