# Supplementary figures and images for: A systems-level model reveals that 1,2-Propanediol utilization microcompartments enhance pathway flux through intermediate sequestration
Source: PLoS Comput Biol. 2017 May 5;13(5):e1005525. doi: 10.1371/journal.pcbi.1005525 (PMC5438192; doi:10.1371/journal.pcbi.1005525)

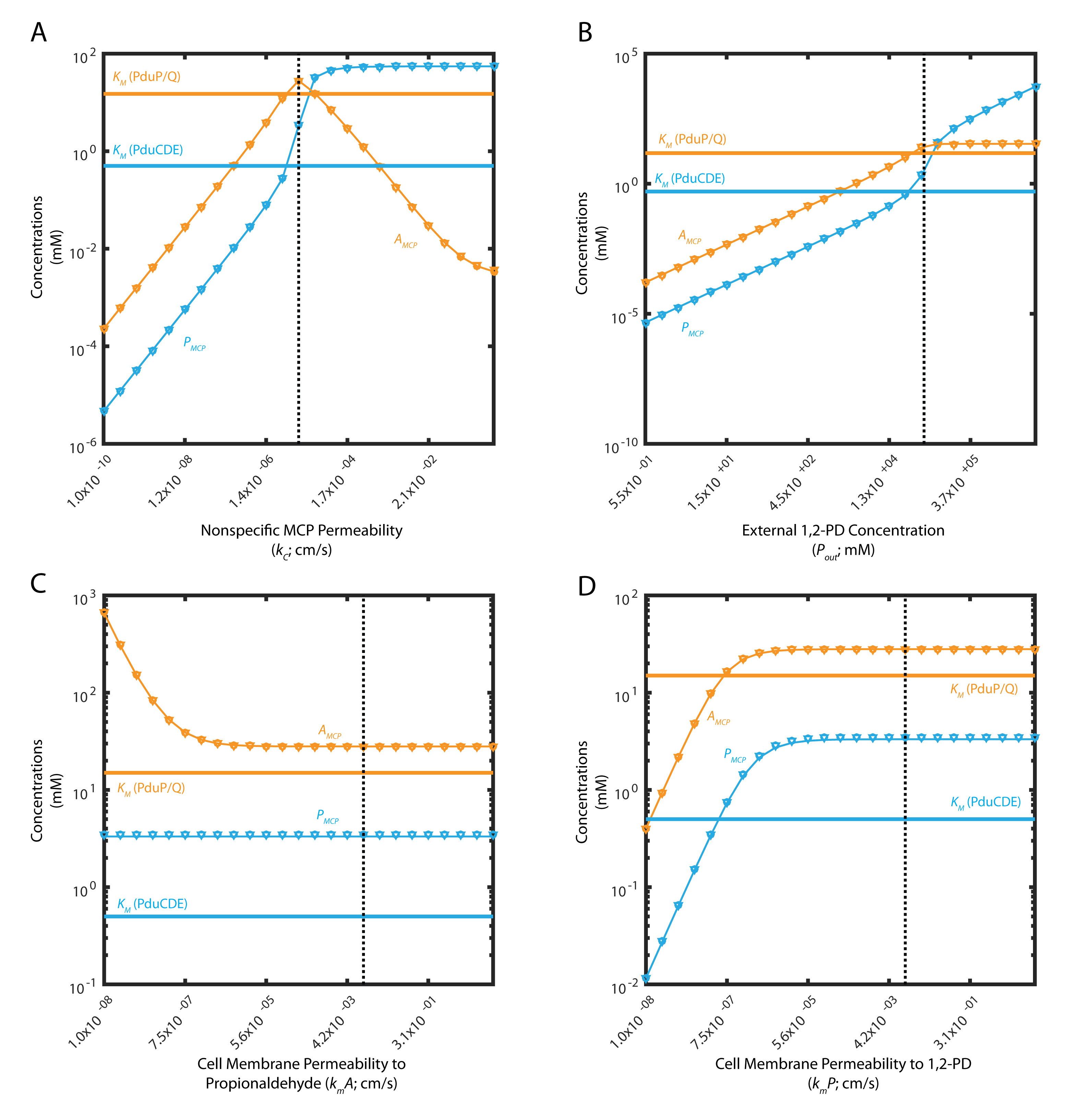

Supplement: S1 Fig — The baseline parameter values are shown with a black dashed line. The KM of the PduCDE and PduP/Q enzymes are plotted in blue and orange lines, respectively. (TIF) [file pcbi.1005525.s002.tif]

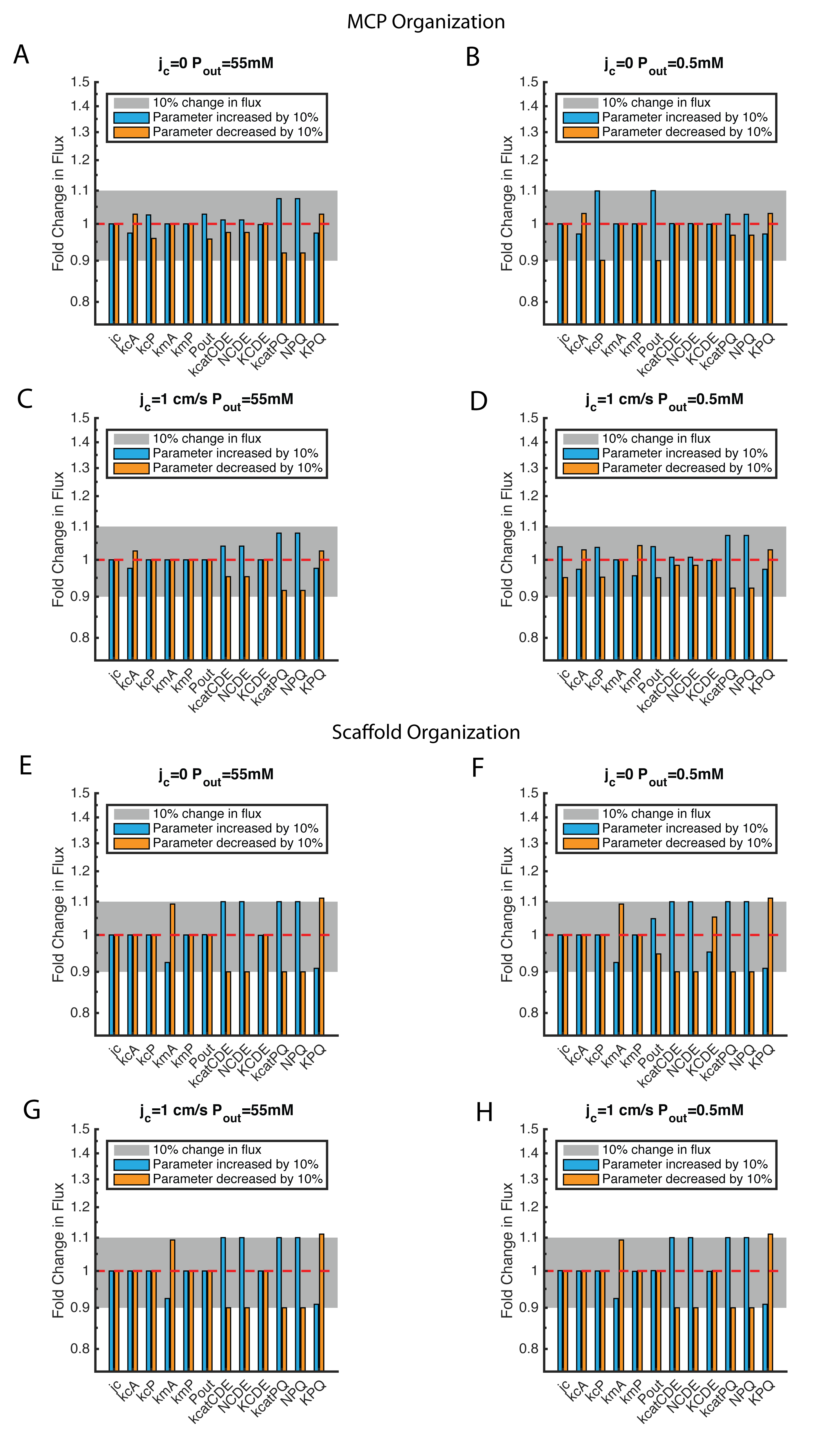

Supplement: S2 Fig — Plotted is the relative flux after the perturbation as compared to the flux using the initial parameters. MCP organization is shown in (A), (B), (C), and (D); scaffold organization is shown in (E), (F), (G), and (H). (A), (C), (E), and (G): Pout = 55 mM. (B), (D), (F), and (H): Pout = 0.5 mM. (A), (B), (E), and (F): no active transport of 1,2-PD. (C), (D), (G), and (H): jc = 1 cm/s. Orange bars indicate the change in flux upon a 10% decrease in the indicated parameter; blue bars indicate the change in flux upon a 10% increase in the indicated parameter. Grey box indicates a 10% change in the relative flux. Red dashed line indicates the flux based on the initial parameters. (TIF) [file pcbi.1005525.s003.tif]

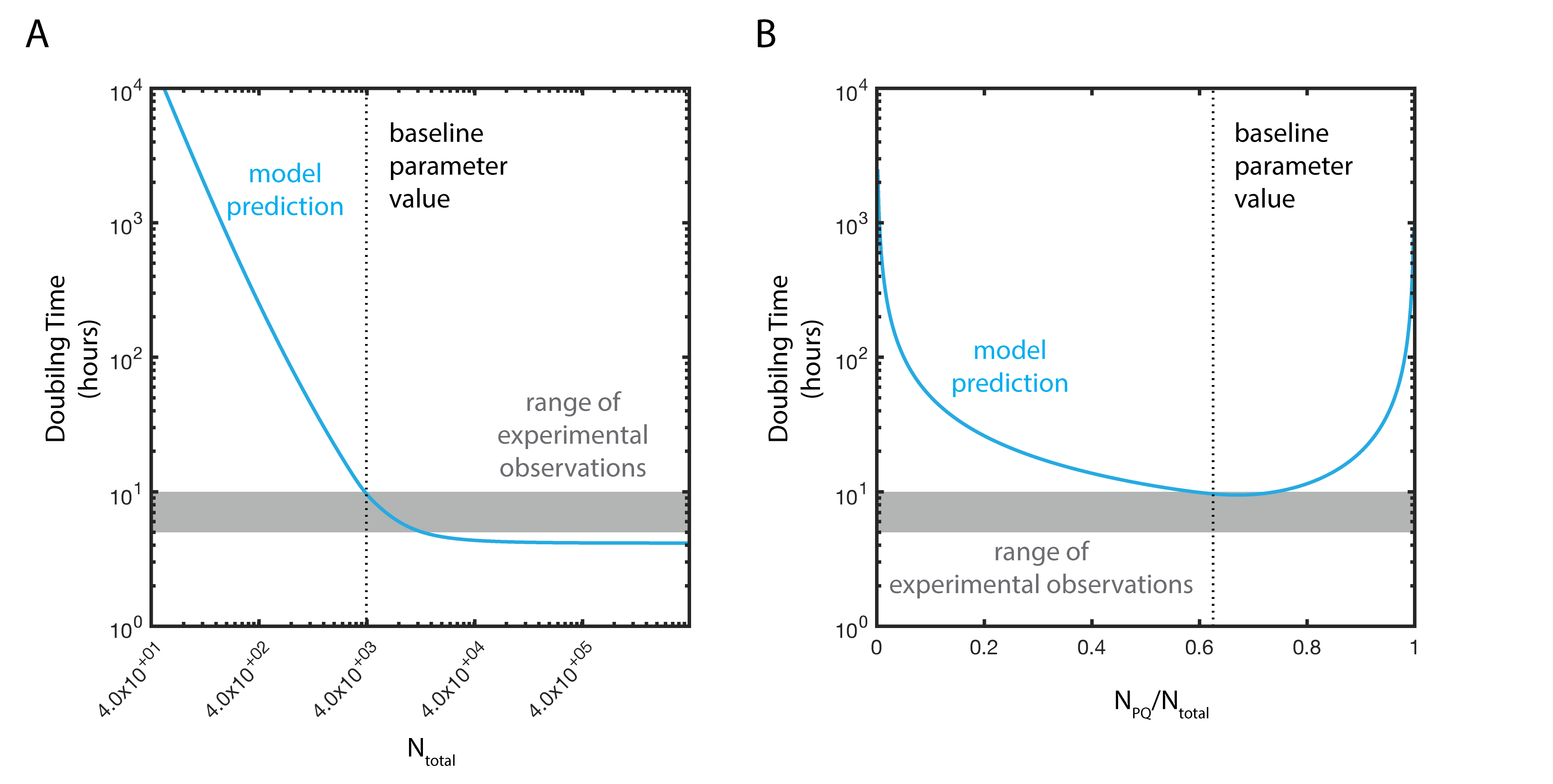

Supplement: S3 Fig — Model predictions are shown in blue; observed doubling times for experiment are shown by the grey shaded area. The experimentally observed Ntotal and NPQNtotal values are shown by black dashed lines in (A) and (B), respectively. NPQNtotal is held constant in (A) and Ntotal is held constant in (B). (TIF) [file pcbi.1005525.s004.tif]

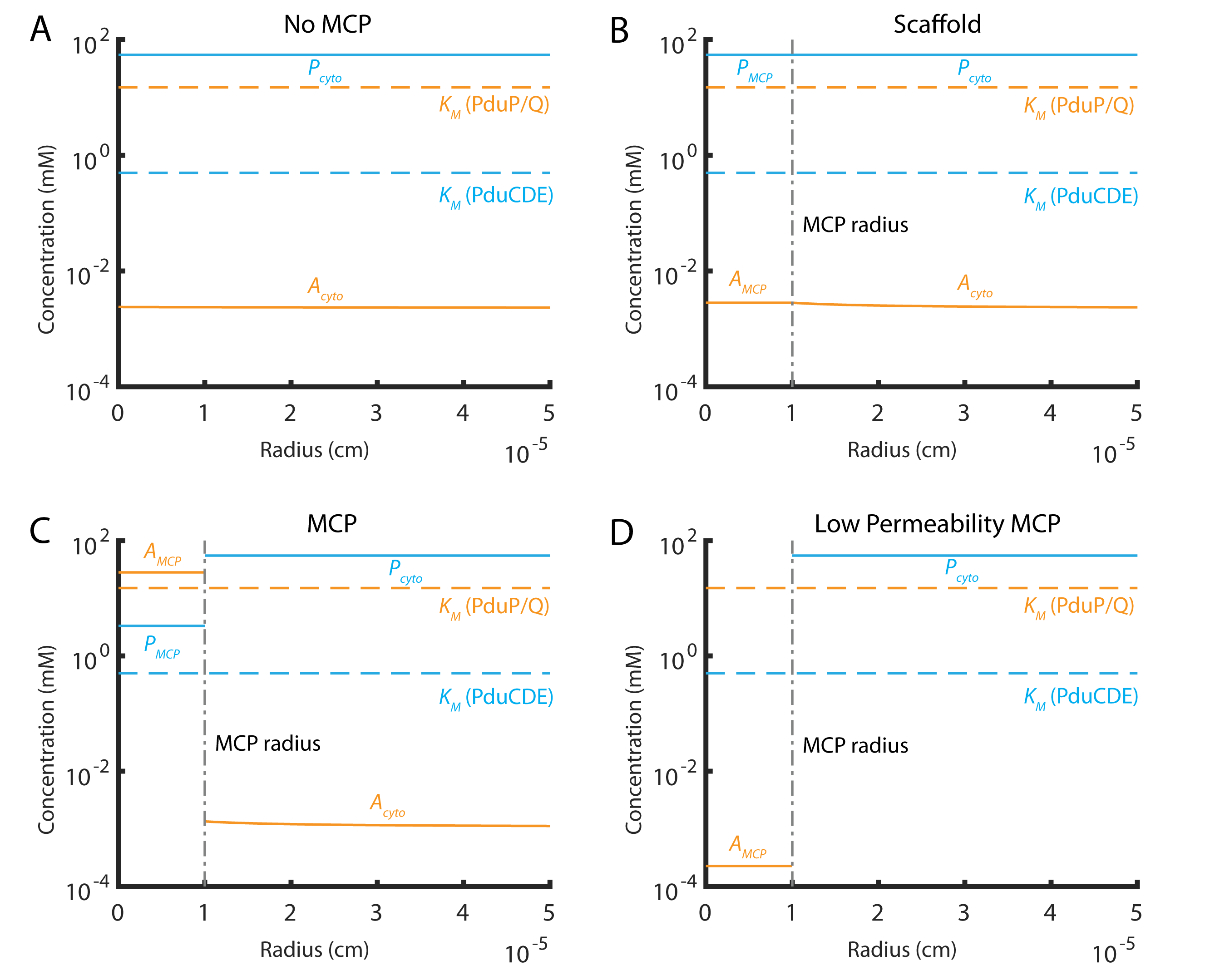

Supplement: S4 Fig — 1,2-PD in the MCP (PMCP) and in the cytosol (Pcyto) are plotted in blue and propionaldehyde in the MCP (AMCP) and in the cytosol (Acyto) in orange. The KM of the PduCDE and PduP/Q enzymes are plotted in blue and orange dashed lines, respectively. (TIF) [file pcbi.1005525.s005.tif]

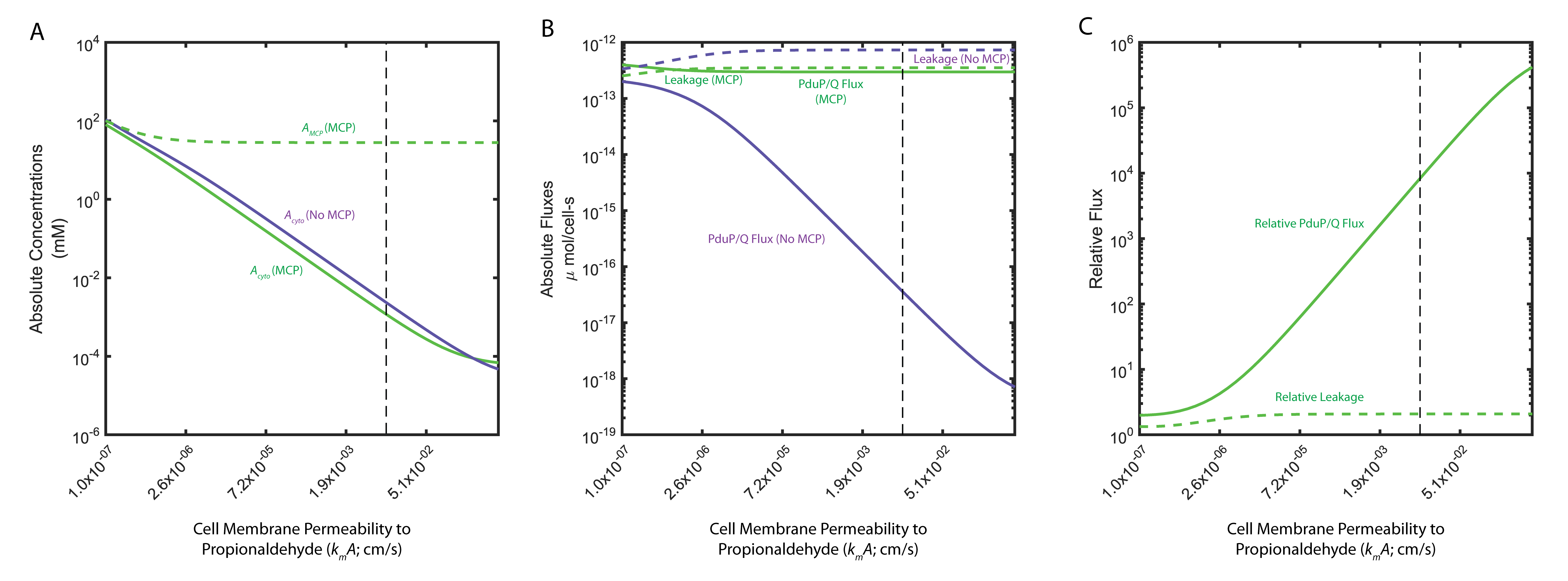

Supplement: S5 Fig — The baseline kmA value is shown with a black dashed line. (TIF) [file pcbi.1005525.s006.tif]

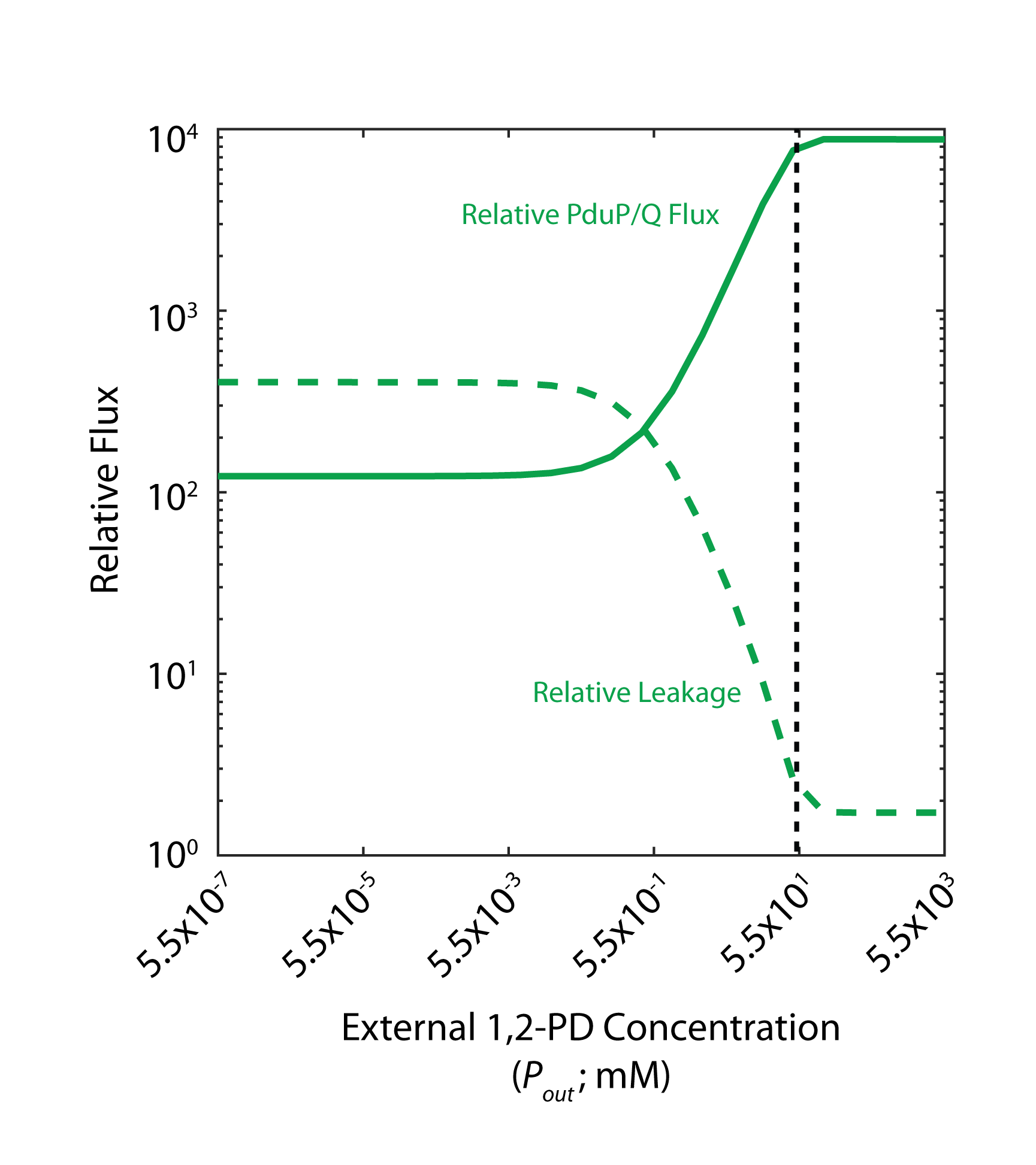

Supplement: S6 Fig — The baseline external 1,2-PD concentration is shown with a black dashed line. (TIF) [file pcbi.1005525.s007.tif]

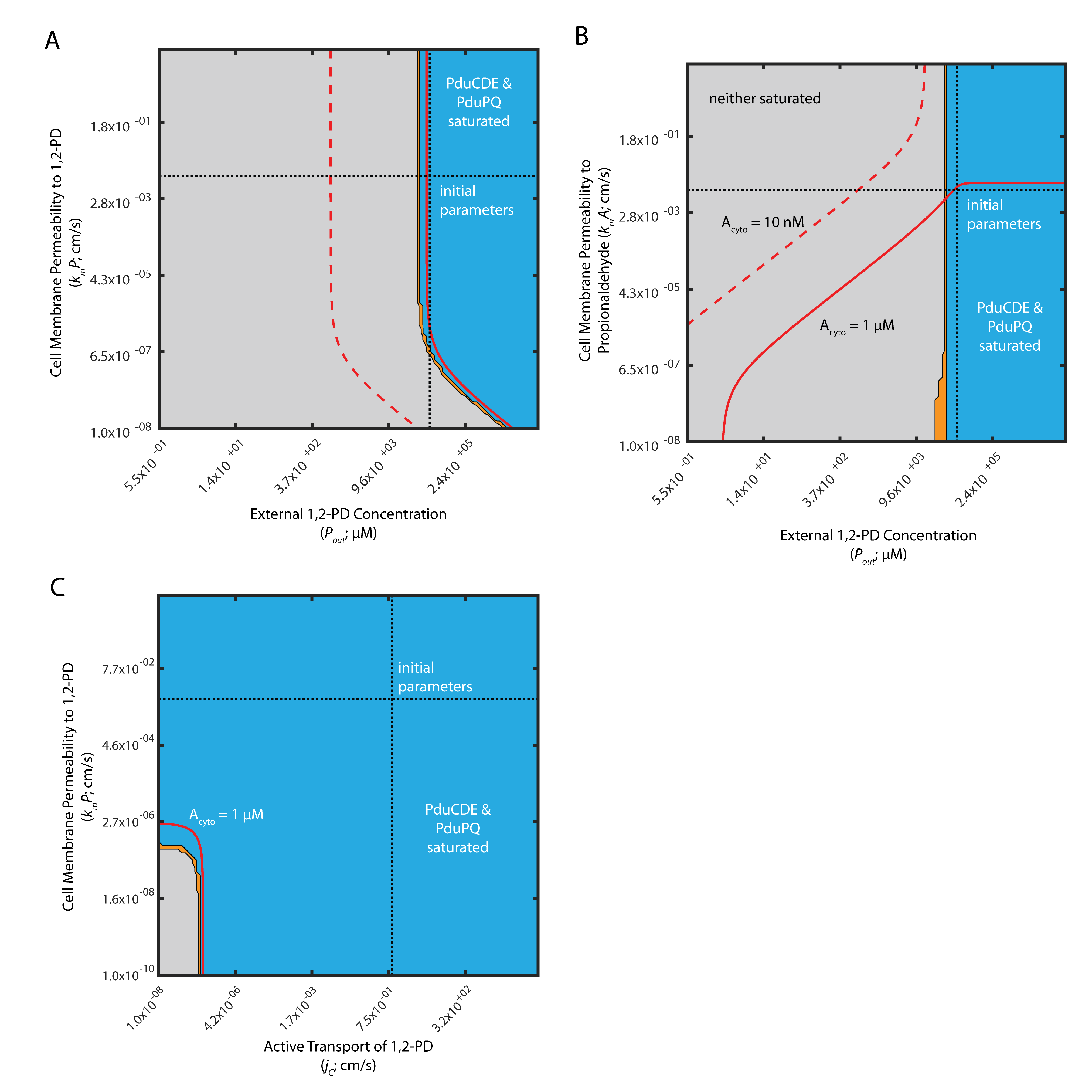

Supplement: S7 Fig — Regions of saturation (concentration of substrate > KM of the appropriate enzyme) are plotted in blue when PduCDE and PduP/Q are saturated, orange when only PduCDE is saturated, and in grey when neither enzyme is saturated. Red solid lines indicate when Acyto is 1 μM; red dashed lines indicate when Acyto is 10 nM. Black dashed lines indicate the baseline parameter values used in the model of the Pdu MCP. (TIF) [file pcbi.1005525.s008.tif]

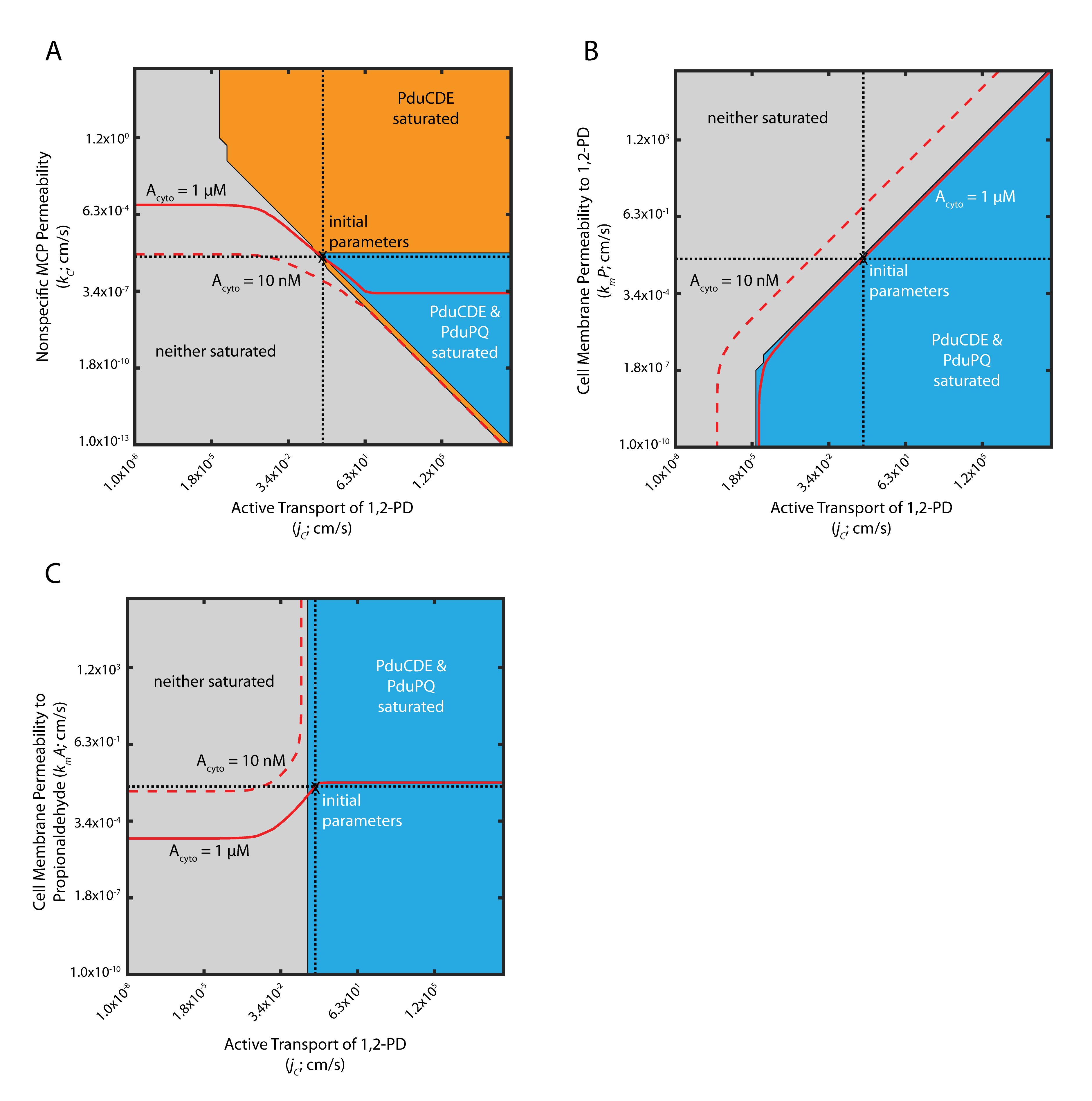

Supplement: S8 Fig — Regions of saturation (concentration of substrate > KM of the appropriate enzyme) are plotted in blue when both enzymes are saturate, orange when only PduCDE is saturated, and in grey when neither enzyme is saturated. Red solid lines indicate when Acyto is 1 μM; red dashed lines indicate when Acyto is 10 nM. Black dashed lines indicate the baseline parameter values used in the model of the Pdu MCP. (TIF) [file pcbi.1005525.s009.tif]

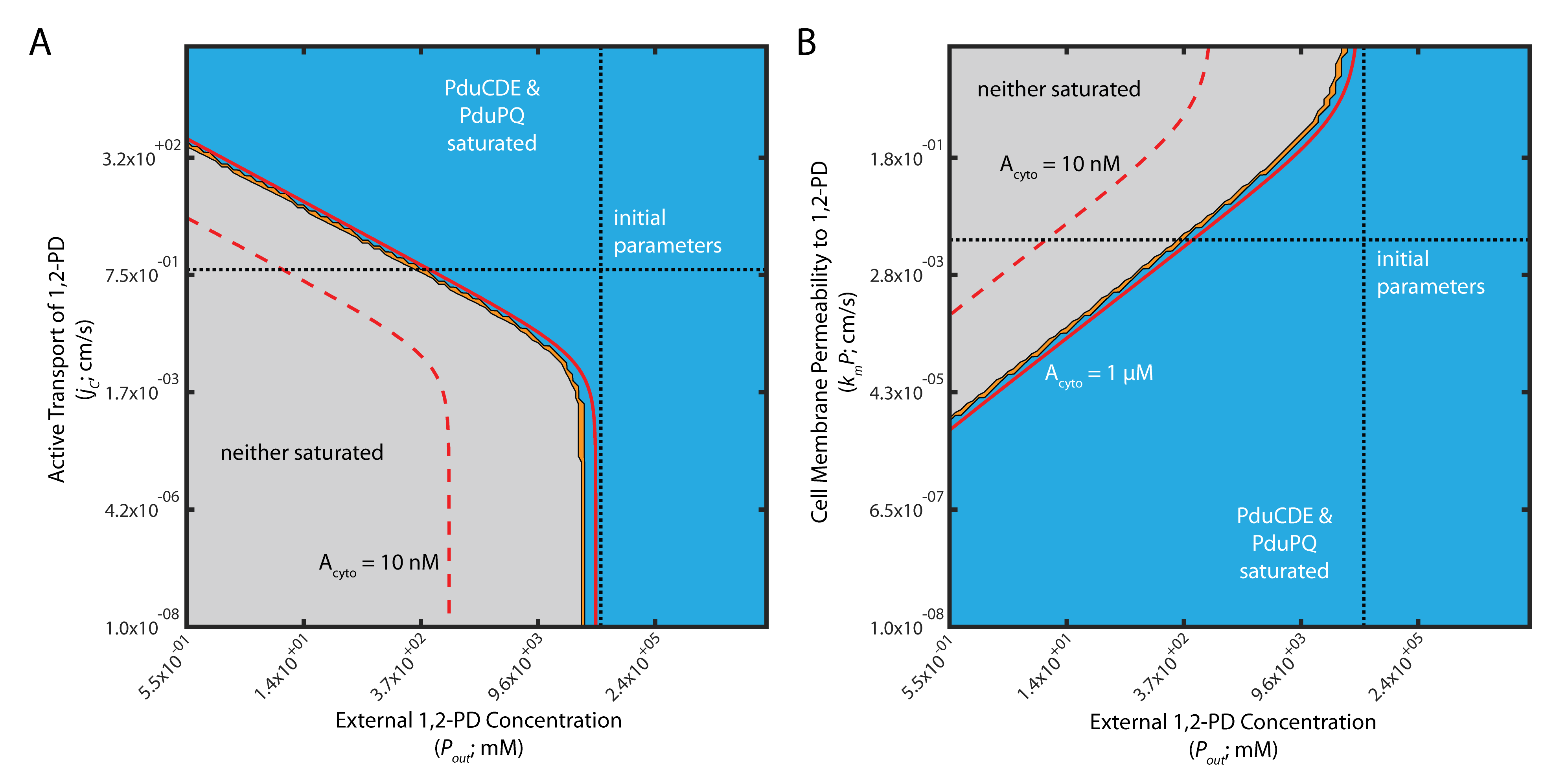

Supplement: S9 Fig — Regions of saturation (concentration of substrate > KM of the appropriate enzyme) are plotted in blue when both enzymes are saturate, orange when only PduCDE is saturated, and in grey when neither enzyme is saturated. Red solid lines indicate when Acyto is 1 μM; red dashed lines indicate Acyto is 10 nM. Black dashed lines indicate the baseline parameter values used in the model of the Pdu MCP. (TIF) [file pcbi.1005525.s010.tif]

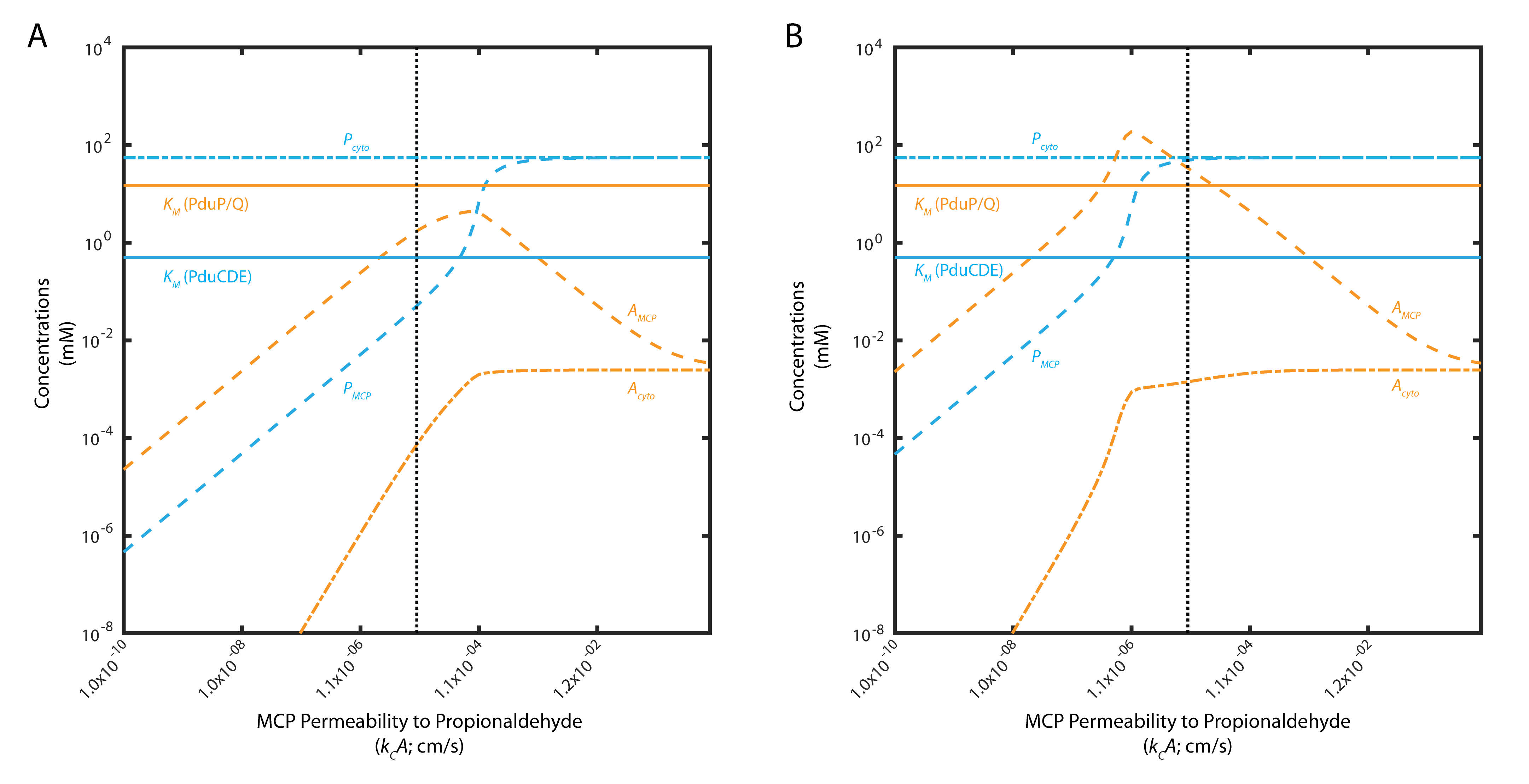

Supplement: S10 Fig — KM of PduCDE and PduP/Q are shown as solid lines. The baseline permeabilities are shown with a black dashed line. (TIF) [file pcbi.1005525.s011.tif]

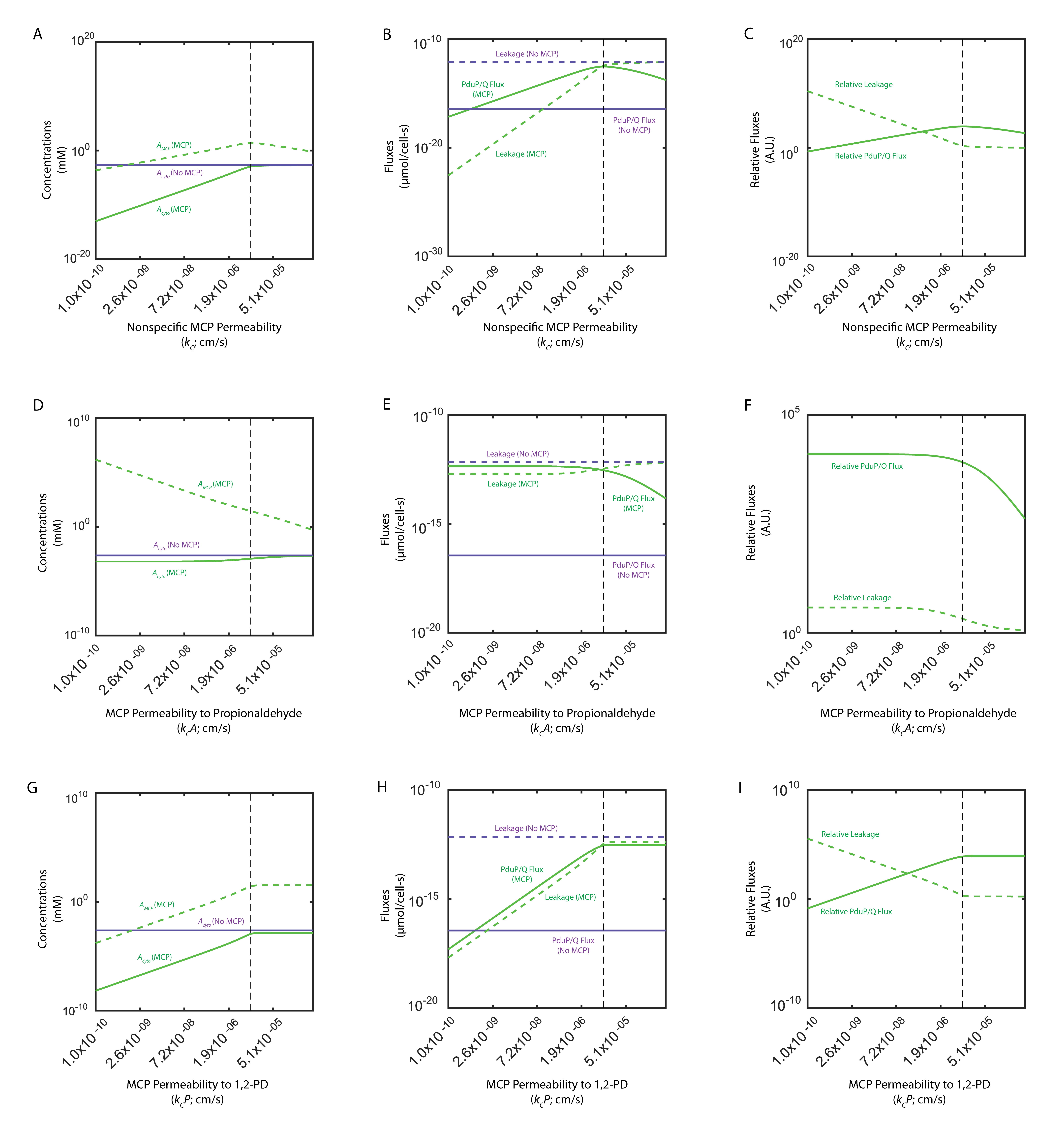

Supplement: S11 Fig — As a function of (A,B,C) kc=kcA=kcP; (D,E,F) kcA; and (G,H,I) kcP. The baseline permeabilities are shown with a black dashed line. (TIF) [file pcbi.1005525.s012.tif]

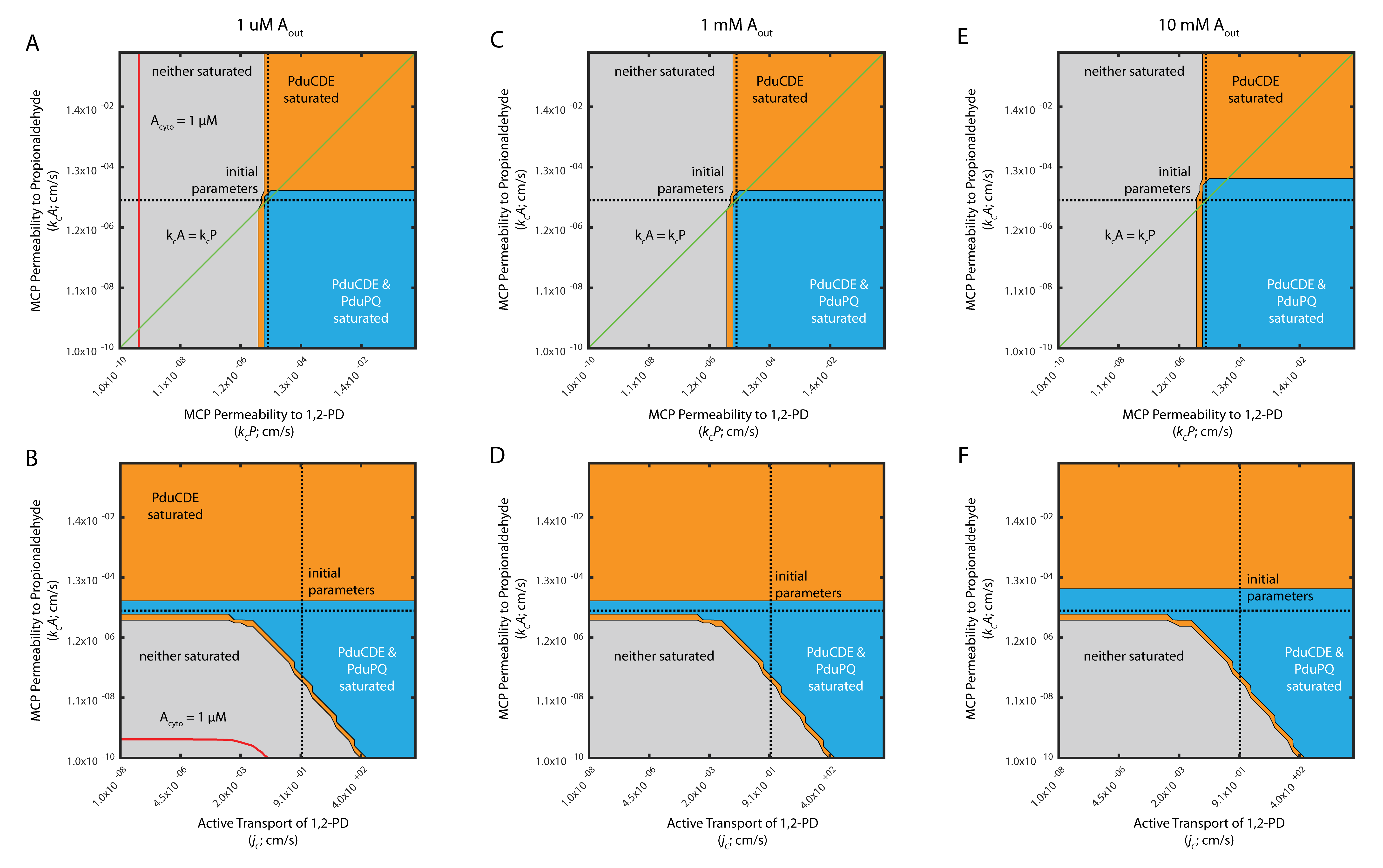

Supplement: S12 Fig — Regions of saturation (concentration of substrate > KM of the appropriate enzyme) are plotted in blue when both enzymes are saturated, orange when only PduCDE is saturated, and in grey when neither enzyme is saturated. Red solid lines indicate when Acyto is 1 μM; red dashed lines indicate when Acyto is 10 nM. Black dashed lines indicate the baseline parameter values used in the model of the Pdu MCP. Green line in (A, C, E) indicates when kcA=kcP. (TIF) [file pcbi.1005525.s013.tif]

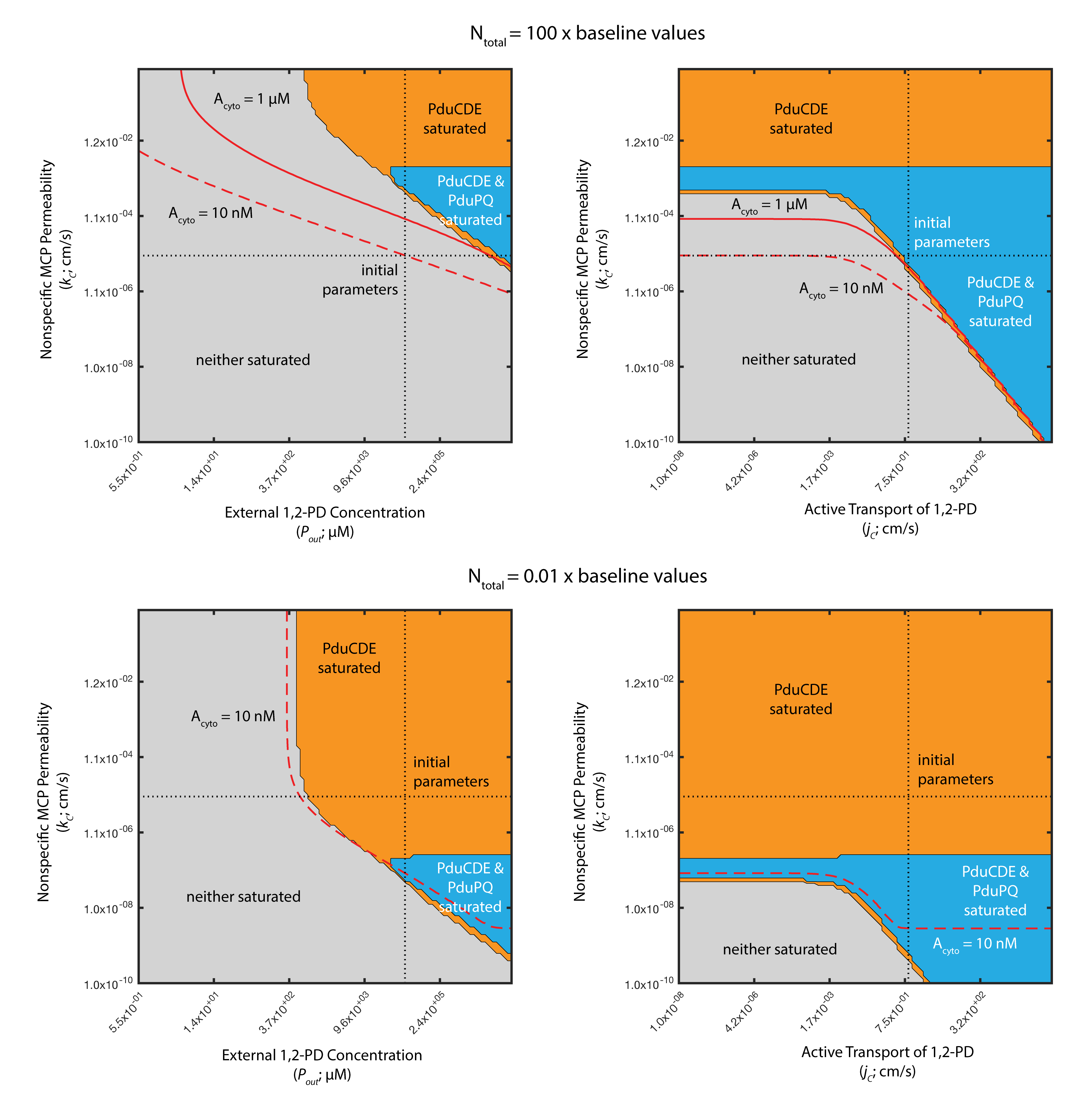

Supplement: S13 Fig — Regions of saturation (concentration of substrate > KM of the appropriate enzyme) are plotted in blue when both enzymes are saturated, orange when only PduCDE is saturated, and in grey when neither enzyme is saturated. Red solid lines indicate when Acyto is 1 μM; red dashed lines indicate when Acyto is 10 nM. Black dashed lines indicate the baseline parameter values used in the model of the Pdu MCP. (TIF) [file pcbi.1005525.s014.tif]
